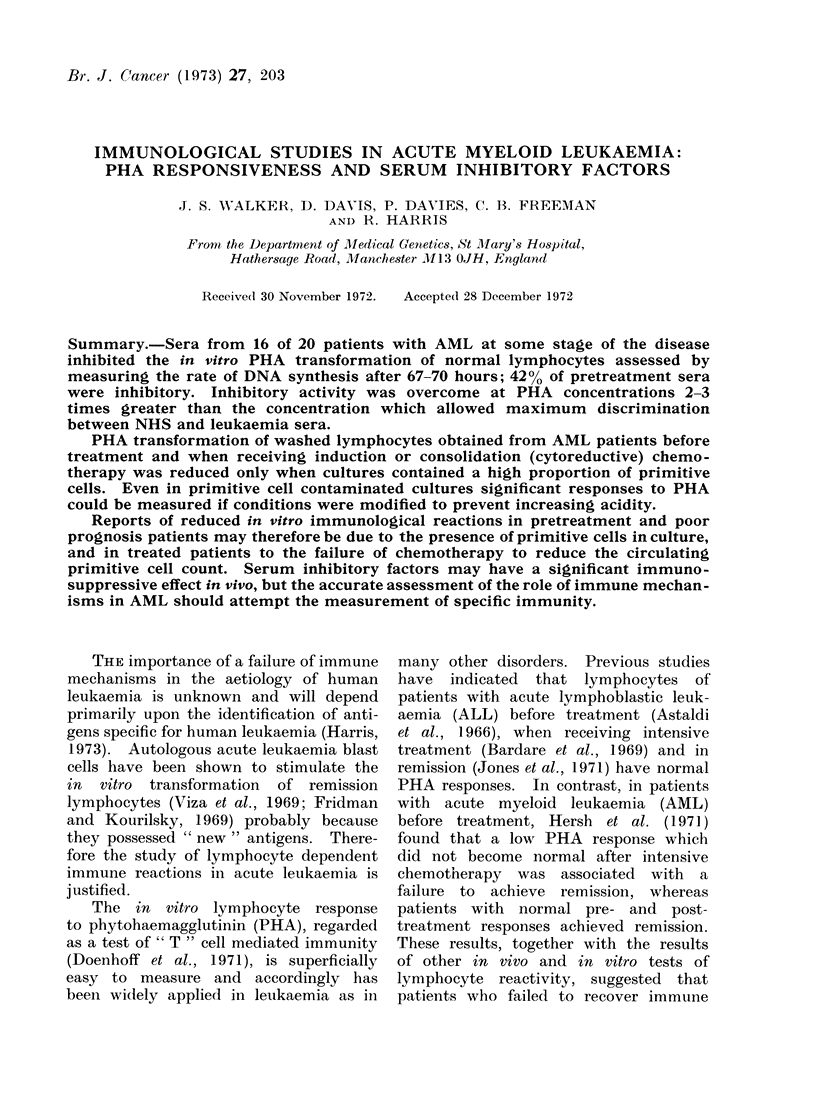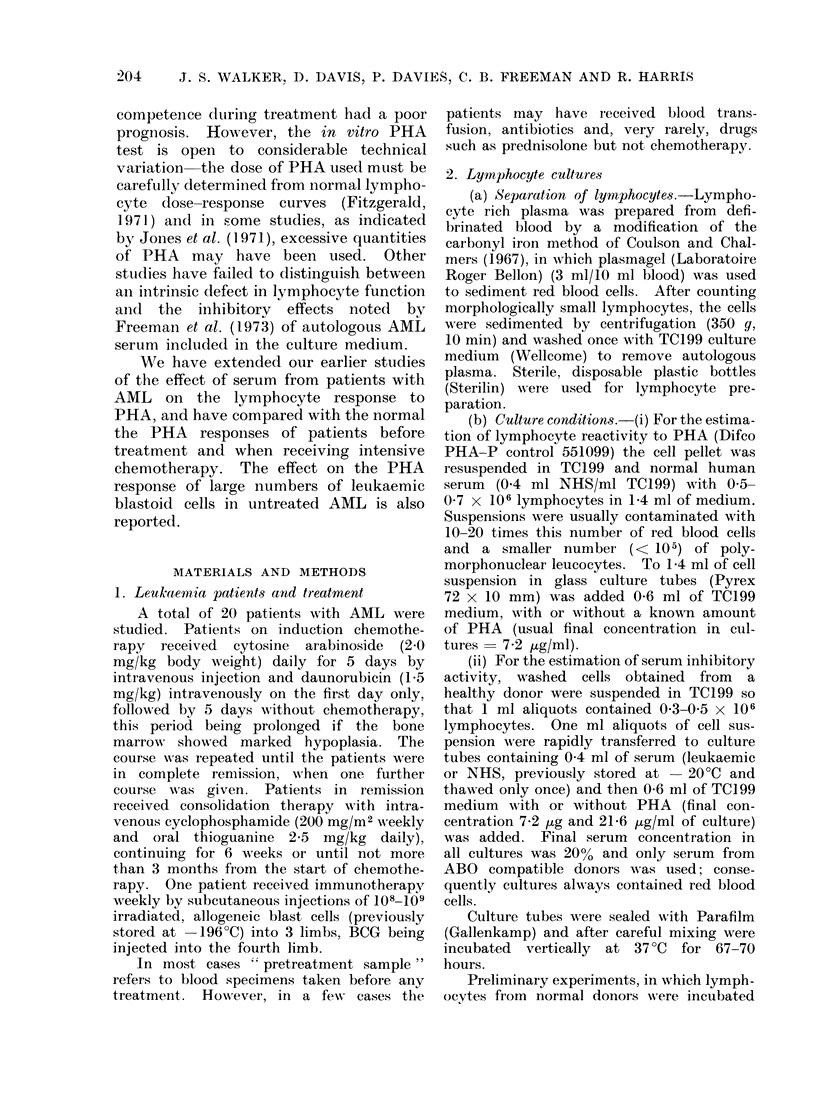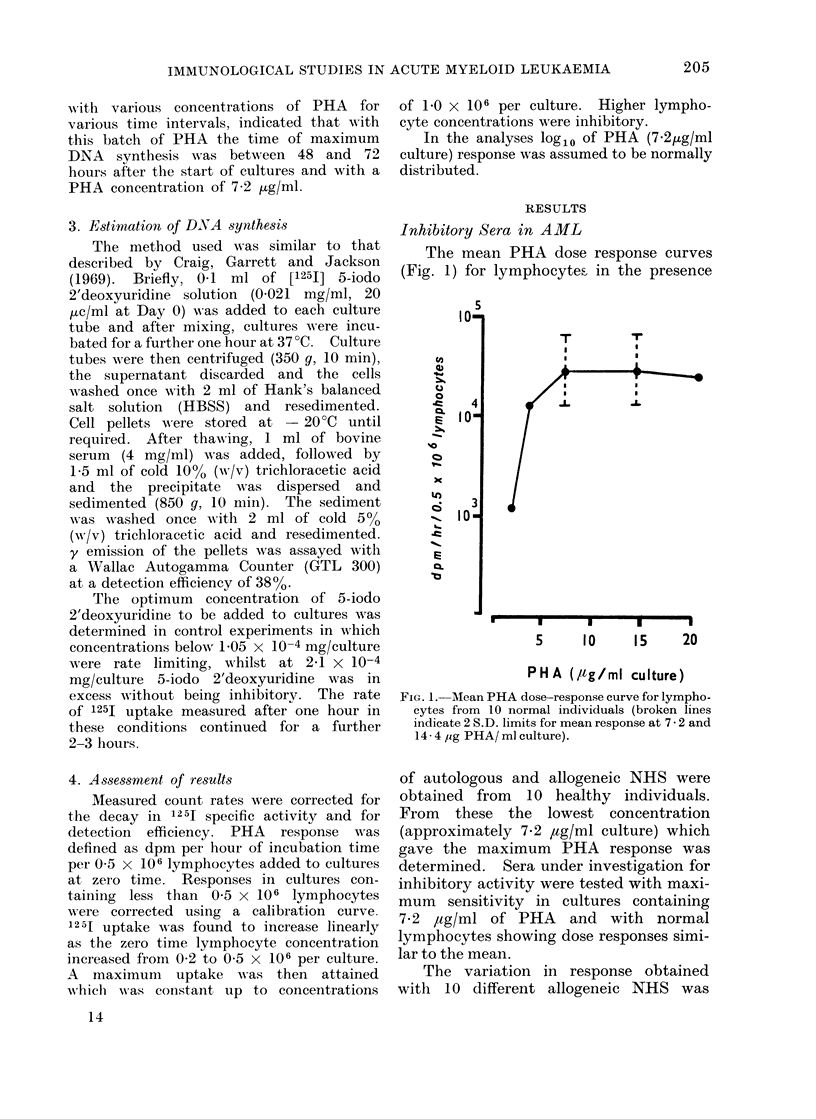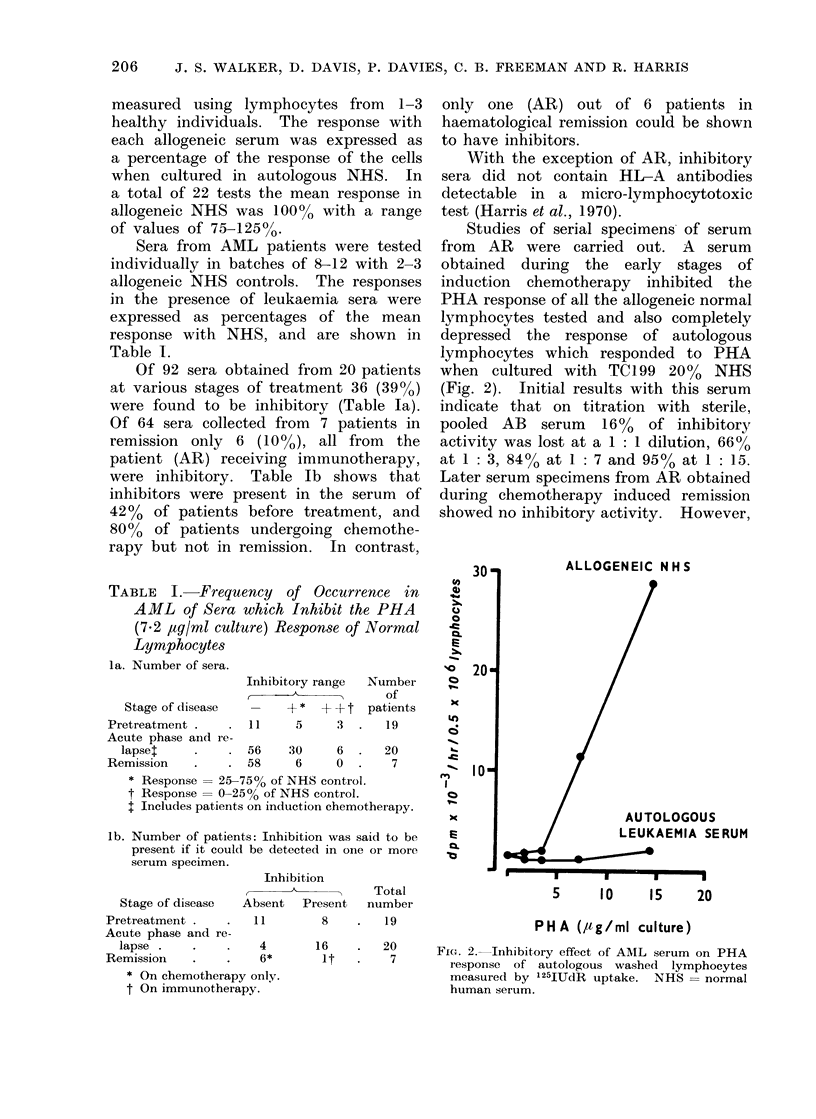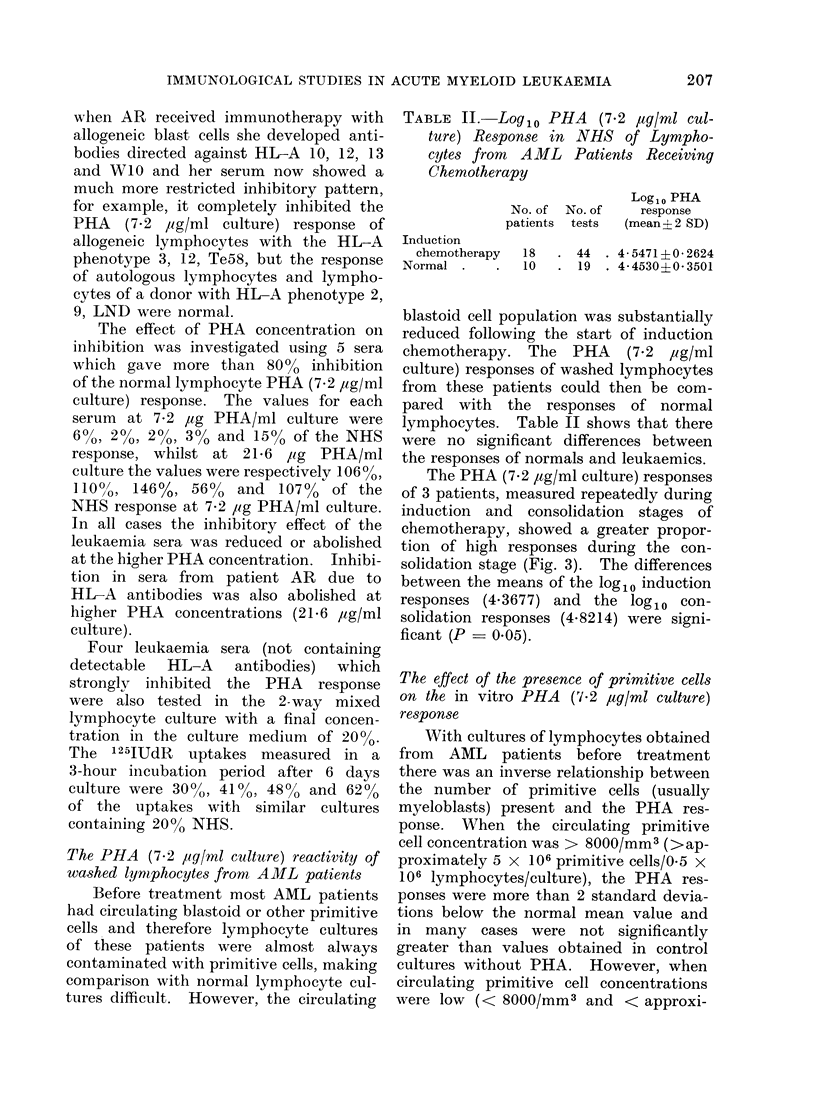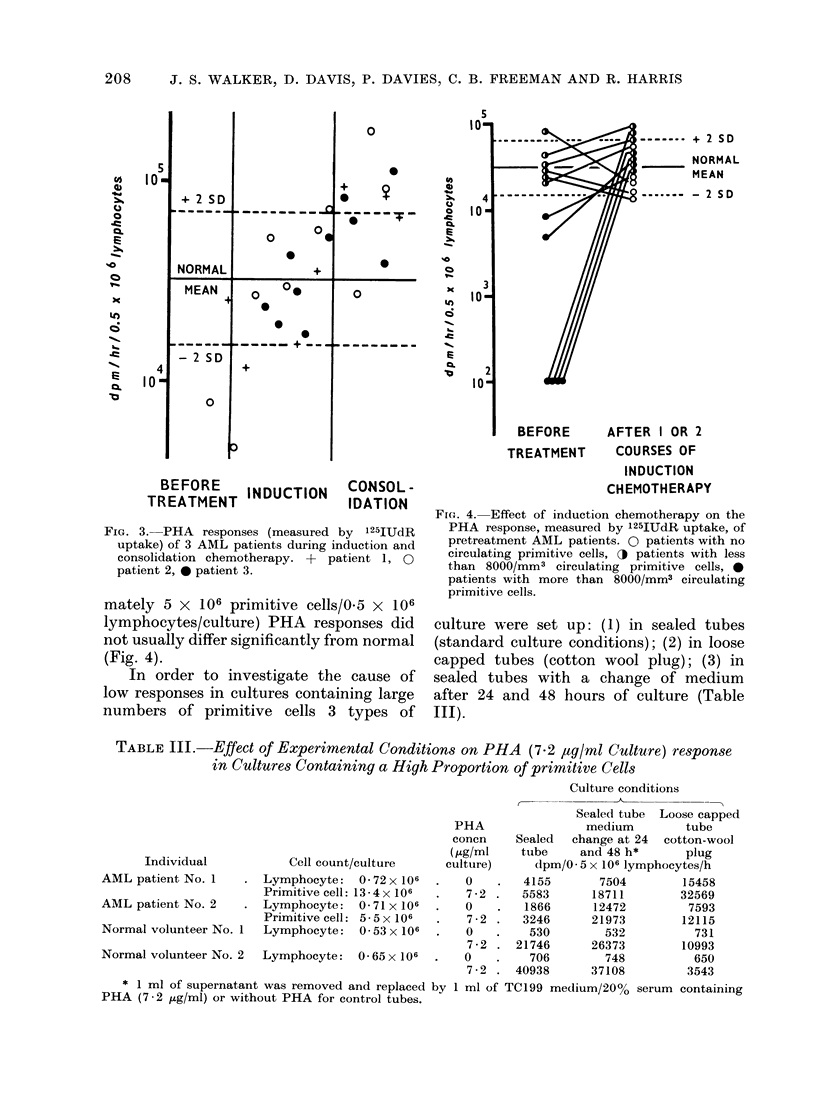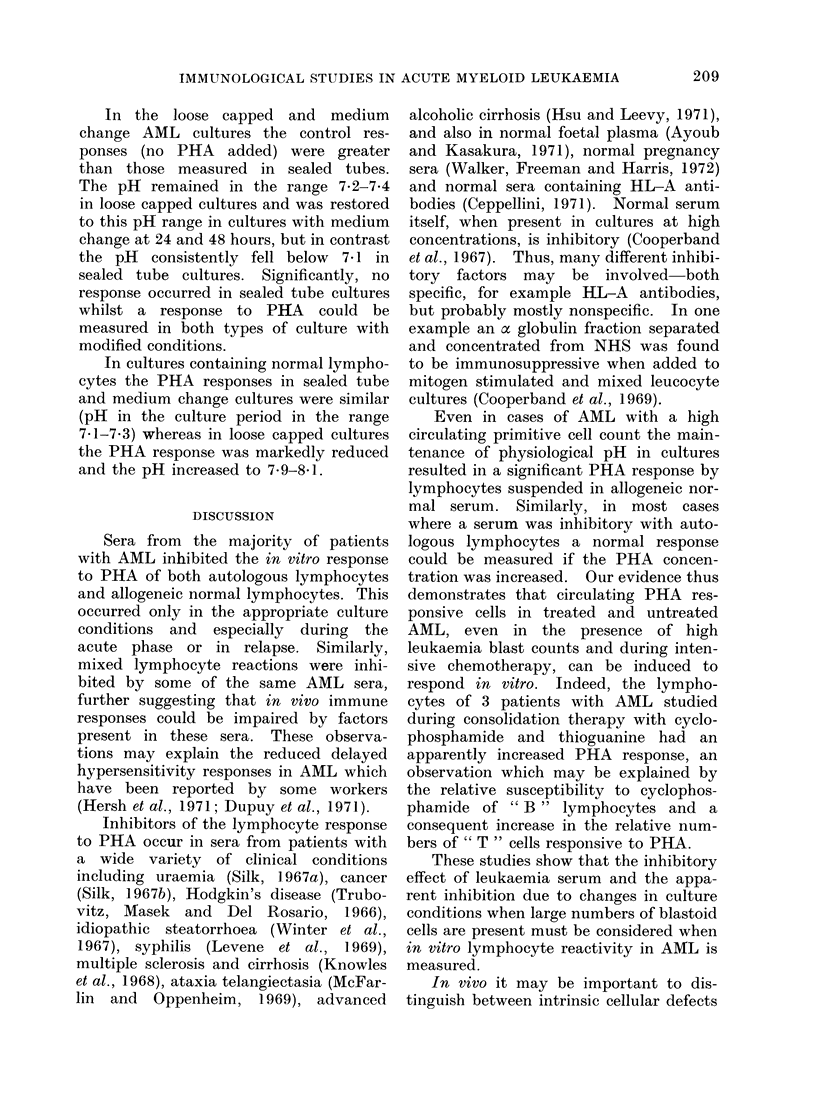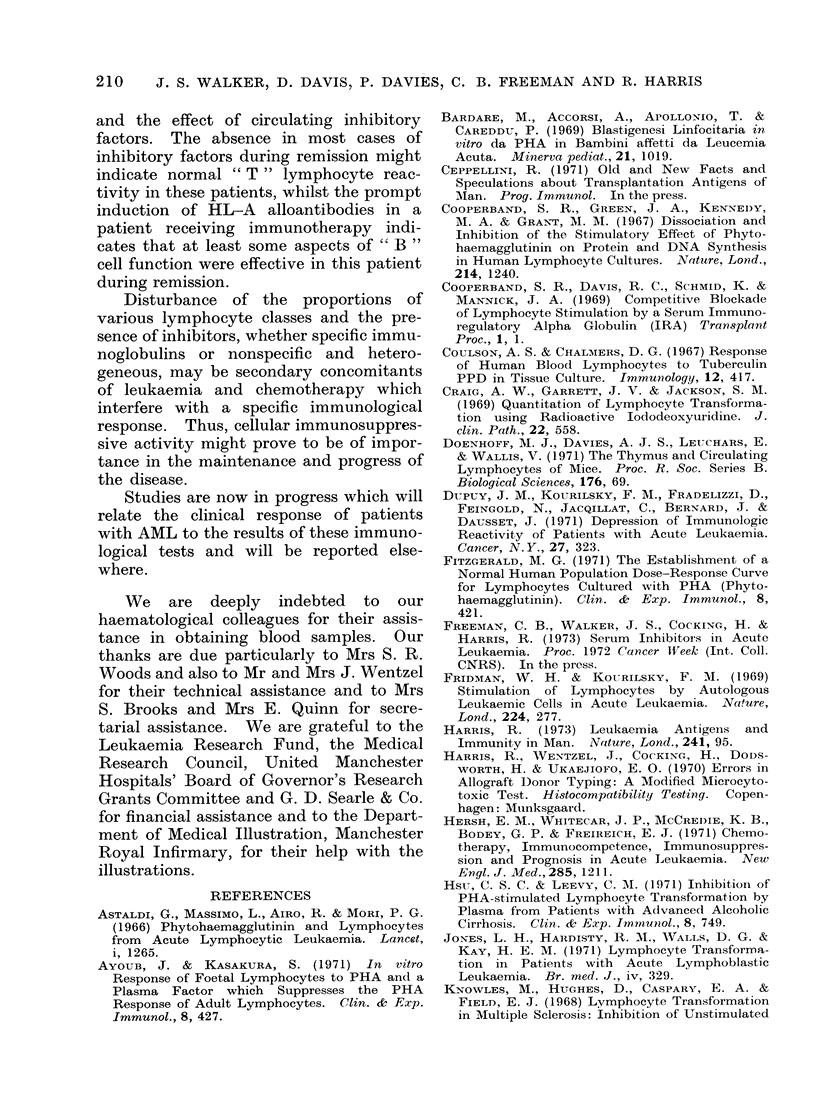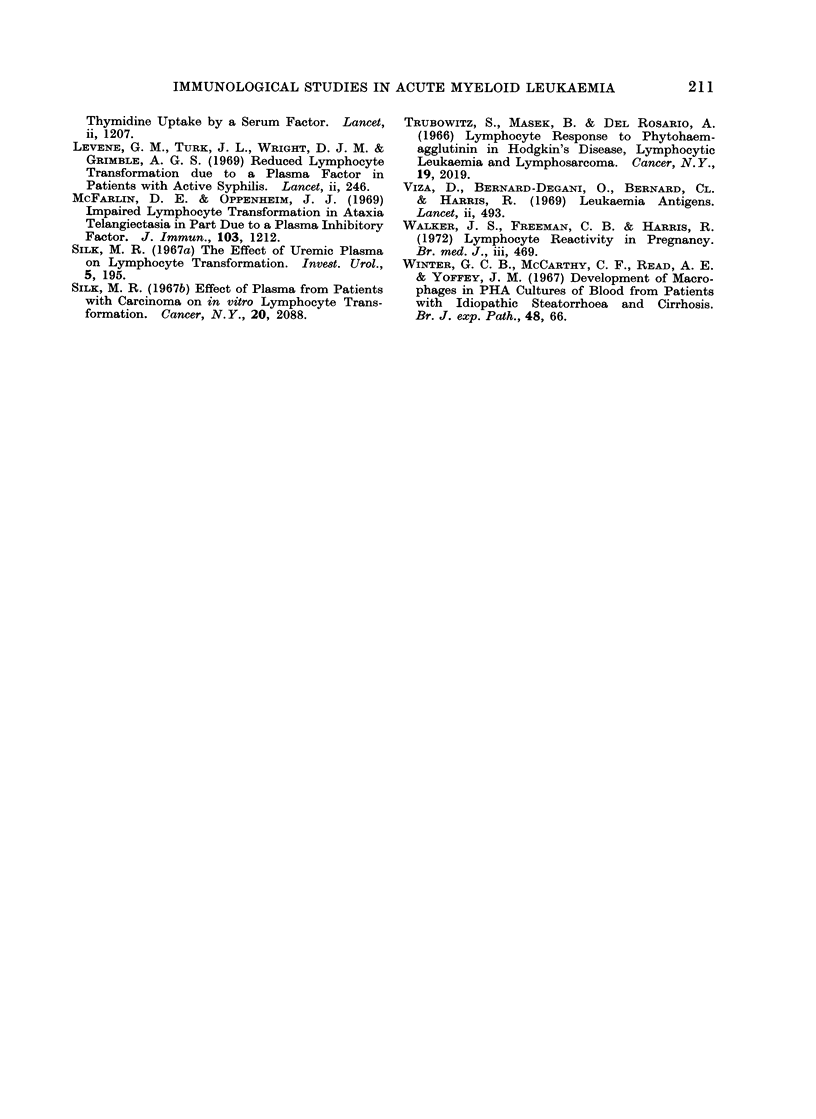# Immunological Studies in Acute Myeloid Leukaemia: PHA Responsiveness and Serum Inhibitory Factors

**DOI:** 10.1038/bjc.1973.25

**Published:** 1973-03

**Authors:** J. S. Walker, D. Davis, P. Davies, C. B. Freeman, R. Harris

## Abstract

Sera from 16 of 20 patients with AML at some stage of the disease inhibited the *in vitro* PHA transformation of normal lymphocytes assessed by measuring the rate of DNA synthesis after 67-70 hours; 42% of pretreatment sera were inhibitory. Inhibitory activity was overcome at PHA concentrations 2-3 times greater than the concentration which allowed maximum discrimination between NHS and leukaemia sera.

PHA transformation of washed lymphocytes obtained from AML patients before treatment and when receiving induction or consolidation (cytoreductive) chemotherapy was reduced only when cultures contained a high proportion of primitive cells. Even in primitive cell contaminated cultures significant responses to PHA could be measured if conditions were modified to prevent increasing acidity.

Reports of reduced *in vitro* immunological reactions in pretreatment and poor prognosis patients may therefore be due to the presence of primitive cells in culture, and in treated patients to the failure of chemotherapy to reduce the circulating primitive cell count. Serum inhibitory factors may have a significant immunosuppressive effect *in vivo,* but the accurate assessment of the role of immune mechanisms in AML should attempt the measurement of specific immunity.


*Br. J. Cancer* (1973) **27**, 203

### IMMUNOLOGICAL STUDIES IN ACUTE MYELOID LEUKAEMIA: PHA RESPONSIVENESS AND SERUM INHIBITORY FACTORS

J. S. WALKER, D. DAVIS, P. DAVIES, C. B. FREEMAN AND R. HARRIS

*From the Department of Medical Genetics, St Mary's Hospital, Hathersage Road, Manchester M13 0JH, England*

Received 30 November 1972. Accepted 28 December 1972

**Summary.—Sera from 16 of 20 patients with AML at some stage of the disease inhibited the *in vitro* PHA transformation of normal lymphocytes assessed by measuring the rate of DNA synthesis after 67–70 hours; 42% of pretreatment sera were inhibitory. Inhibitory activity was overcome at PHA concentrations 2–3 times greater than the concentration which allowed maximum discrimination between NHS and leukaemia sera.**

**PHA transformation of washed lymphocytes obtained from AML patients before treatment and when receiving induction or consolidation (cytoreductive) chemotherapy was reduced only when cultures contained a high proportion of primitive cells. Even in primitive cell contaminated cultures significant responses to PHA could be measured if conditions were modified to prevent increasing acidity.**

**Reports of reduced *in vitro* immunological reactions in pretreatment and poor prognosis patients may therefore be due to the presence of primitive cells in culture, and in treated patients to the failure of chemotherapy to reduce the circulating primitive cell count. Serum inhibitory factors may have a significant immunosuppressive effect *in vivo*, but the accurate assessment of the role of immune mechanisms in AML should attempt the measurement of specific immunity.**

THE importance of a failure of immune mechanisms in the aetiology of human leukaemia is unknown and will depend primarily upon the identification of antigens specific for human leukaemia (Harris, 1973). Autologous acute leukaemia blast cells have been shown to stimulate the *in vitro* transformation of remission lymphocytes (Viza *et al.*, 1969; Fridman and Kourilsky, 1969) probably because they possessed "new" antigens. Therefore the study of lymphocyte dependent immune reactions in acute leukaemia is justified.

The *in vitro* lymphocyte response to phytohaemagglutinin (PHA), regarded as a test of "T" cell mediated immunity (Doenhoff *et al.*, 1971), is superficially easy to measure and accordingly has been widely applied in leukaemia as in many other disorders. Previous studies have indicated that lymphocytes of patients with acute lymphoblastic leukaemia (ALL) before treatment (Astaldi *et al.*, 1966), when receiving intensive treatment (Bardare *et al.*, 1969) and in remission (Jones *et al.*, 1971) have normal PHA responses. In contrast, in patients with acute myeloid leukaemia (AML) before treatment, Hersh *et al.* (1971) found that a low PHA response which did not become normal after intensive chemotherapy was associated with a failure to achieve remission, whereas patients with normal pre- and post-treatment responses achieved remission. These results, together with the results of other *in vivo* and *in vitro* tests of lymphocyte reactivity, suggested that patients who failed to recover immune competence during treatment had a poor prognosis. However, the *in vitro* PHA test is open to considerable technical variation—the dose of PHA used must be carefully determined from normal lymphocyte dose–response curves (Fitzgerald, 1971) and in some studies, as indicated by Jones *et al.* (1971), excessive quantities of PHA may have been used. Other studies have failed to distinguish between an intrinsic defect in lymphocyte function and the inhibitory effects noted by Freeman *et al.* (1973) of autologous AML serum included in the culture medium.

We have extended our earlier studies of the effect of serum from patients with AML on the lymphocyte response to PHA, and have compared with the normal the PHA responses of patients before treatment and when receiving intensive chemotherapy. The effect on the PHA response of large numbers of leukaemic blastoid cells in untreated AML is also reported.

### MATERIALS AND METHODS

1. *Leukaemia patients and treatment*

A total of 20 patients with AML were studied. Patients on induction chemotherapy received cytosine arabinoside (2·0 mg/kg body weight) daily for 5 days by intravenous injection and daunorubicin (1·5 mg/kg) intravenously on the first day only, followed by 5 days without chemotherapy, this period being prolonged if the bone marrow showed marked hypoplasia. The course was repeated until the patients were in complete remission, when one further course was given. Patients in remission received consolidation therapy with intravenous cyclophosphamide (200 mg/m$^2$ weekly and oral thioguanine 2·5 mg/kg daily), continuing for 6 weeks or until not more than 3 months from the start of chemotherapy. One patient received immunotherapy weekly by subcutaneous injections of $10^8$–$10^9$ irradiated, allogeneic blast cells (previously stored at $-196$°C) into 3 limbs, BCG being injected into the fourth limb.

In most cases " pretreatment sample " refers to blood specimens taken before any treatment. However, in a few cases the patients may have received blood transfusion, antibiotics and, very rarely, drugs such as prednisolone but not chemotherapy.

2. *Lymphocyte cultures*

(a) *Separation of lymphocytes.*—Lymphocyte rich plasma was prepared from defibrinated blood by a modification of the carbonyl iron method of Coulson and Chalmers (1967), in which plasmagel (Laboratoire Roger Bellon) (3 ml/10 ml blood) was used to sediment red blood cells. After counting morphologically small lymphocytes, the cells were sedimented by centrifugation (350 *g*, 10 min) and washed once with TC199 culture medium (Wellcome) to remove autologous plasma. Sterile, disposable plastic bottles (Sterilin) were used for lymphocyte preparation.

(b) *Culture conditions.*—(i) For the estimation of lymphocyte reactivity to PHA (Difco PHA-P control 551099) the cell pellet was resuspended in TC199 and normal human serum (0·4 ml NHS/ml TC199) with 0·5–0·7 $\times$ $10^6$ lymphocytes in 1·4 ml of medium. Suspensions were usually contaminated with 10–20 times this number of red blood cells and a smaller number ($< 10^5$) of polymorphonuclear leucocytes. To 1·4 ml of cell suspension in glass culture tubes (Pyrex 72 $\times$ 10 mm) was added 0·6 ml of TC199 medium, with or without a known amount of PHA (usual final concentration in cultures = 7·2 $\mu$g/ml).

(ii) For the estimation of serum inhibitory activity, washed cells obtained from a healthy donor were suspended in TC199 so that 1 ml aliquots contained 0·3–0·5 $\times$ $10^6$ lymphocytes. One ml aliquots of cell suspension were rapidly transferred to culture tubes containing 0·4 ml of serum (leukaemic or NHS, previously stored at $-$ 20°C and thawed only once) and then 0·6 ml of TC199 medium with or without PHA (final concentration 7·2 $\mu$g and 21·6 $\mu$g/ml of culture) was added. Final serum concentration in all cultures was 20% and only serum from ABO compatible donors was used; consequently cultures always contained red blood cells.

Culture tubes were sealed with Parafilm (Gallenkamp) and after careful mixing were incubated vertically at 37°C for 67–70 hours.

Preliminary experiments, in which lymphocytes from normal donors were incubated with various concentrations of PHA for various time intervals, indicated that with this batch of PHA the time of maximum DNA synthesis was between 48 and 72 hours after the start of cultures and with a PHA concentration of 7·2 μg/ml.

3. *Estimation of DNA synthesis*

The method used was similar to that described by Craig, Garrett and Jackson (1969). Briefly, 0·1 ml of [$^{125}$I] 5-iodo 2′deoxyuridine solution (0·021 mg/ml, 20 μc/ml at Day 0) was added to each culture tube and after mixing, cultures were incubated for a further one hour at 37 °C. Culture tubes were then centrifuged (350 *g*, 10 min), the supernatant discarded and the cells washed once with 2 ml of Hank's balanced salt solution (HBSS) and resedimented. Cell pellets were stored at − 20°C until required. After thawing, 1 ml of bovine serum (4 mg/ml) was added, followed by 1·5 ml of cold 10% (w/v) trichloracetic acid and the precipitate was dispersed and sedimented (850 *g*, 10 min). The sediment was washed once with 2 ml of cold 5% (w/v) trichloracetic acid and resedimented. $\gamma$ emission of the pellets was assayed with a Wallac Autogamma Counter (GTL 300) at a detection efficiency of 38%.

The optimum concentration of 5-iodo 2′deoxyuridine to be added to cultures was determined in control experiments in which concentrations below $1{\cdot}05 \times 10^{-4}$ mg/culture were rate limiting, whilst at $2{\cdot}1 \times 10^{-4}$ mg/culture 5-iodo 2′deoxyuridine was in excess without being inhibitory. The rate of $^{125}$I uptake measured after one hour in these conditions continued for a further 2–3 hours.

4. *Assessment of results*

Measured count rates were corrected for the decay in $^{125}$I specific activity and for detection efficiency. PHA response was defined as dpm per hour of incubation time per $0{\cdot}5 \times 10^6$ lymphocytes added to cultures at zero time. Responses in cultures containing less than $0{\cdot}5 \times 10^6$ lymphocytes were corrected using a calibration curve. $^{125}$I uptake was found to increase linearly as the zero time lymphocyte concentration increased from 0·2 to $0{\cdot}5 \times 10^6$ per culture. A maximum uptake was then attained which was constant up to concentrations of $1{\cdot}0 \times 10^6$ per culture. Higher lymphocyte concentrations were inhibitory.

In the analyses $\log_{10}$ of PHA (7·2μg/ml culture) response was assumed to be normally distributed.

### RESULTS

*Inhibitory Sera in AML*

The mean PHA dose response curves (Fig. 1) for lymphocytes in the presence

FIG. 1.—Mean PHA dose–response curve for lymphocytes from 10 normal individuals (broken lines indicate 2 S.D. limits for mean response at 7·2 and 14·4 μg PHA/ ml culture).

of autologous and allogeneic NHS were obtained from 10 healthy individuals. From these the lowest concentration (approximately 7·2 μg/ml culture) which gave the maximum PHA response was determined. Sera under investigation for inhibitory activity were tested with maximum sensitivity in cultures containing 7·2 μg/ml of PHA and with normal lymphocytes showing dose responses similar to the mean.

The variation in response obtained with 10 different allogeneic NHS was measured using lymphocytes from 1–3 healthy individuals. The response with each allogeneic serum was expressed as a percentage of the response of the cells when cultured in autologous NHS. In a total of 22 tests the mean response in allogeneic NHS was 100% with a range of values of 75–125%.

Sera from AML patients were tested individually in batches of 8–12 with 2–3 allogeneic NHS controls. The responses in the presence of leukaemia sera were expressed as percentages of the mean response with NHS, and are shown in Table I.

Of 92 sera obtained from 20 patients at various stages of treatment 36 (39%) were found to be inhibitory (Table Ia). Of 64 sera collected from 7 patients in remission only 6 (10%), all from the patient (AR) receiving immunotherapy, were inhibitory. Table Ib shows that inhibitors were present in the serum of 42% of patients before treatment, and 80% of patients undergoing chemotherapy but not in remission. In contrast, only one (AR) out of 6 patients in haematological remission could be shown to have inhibitors.

With the exception of AR, inhibitory sera did not contain HL–A antibodies detectable in a micro-lymphocytotoxic test (Harris *et al.*, 1970).

Studies of serial specimens of serum from AR were carried out. A serum obtained during the early stages of induction chemotherapy inhibited the PHA response of all the allogeneic normal lymphocytes tested and also completely depressed the response of autologous lymphocytes which responded to PHA when cultured with TC199 20% NHS (Fig. 2). Initial results with this serum indicate that on titration with sterile, pooled AB serum 16% of inhibitory activity was lost at a 1 : 1 dilution, 66% at 1 : 3, 84% at 1 : 7 and 95% at 1 : 15. Later serum specimens from AR obtained during chemotherapy induced remission showed no inhibitory activity. However,

TABLE I.—*Frequency of Occurrence in AML of Sera which Inhibit the PHA (7·2 μg/ml culture) Response of Normal Lymphocytes*

Ia. Number of sera.

| Stage of disease | Inhibitory range – | Inhibitory range +* | Inhibitory range ++† | Number of patients |
|---|---|---|---|---|
| Pretreatment | 11 | 5 | 3 | 19 |
| Acute phase and relapse‡ | 56 | 30 | 6 | 20 |
| Remission | 58 | 6 | 0 | 7 |

* Response = 25–75% of NHS control.
† Response = 0–25% of NHS control.
‡ Includes patients on induction chemotherapy.

Ib. Number of patients: Inhibition was said to be present if it could be detected in one or more serum specimen.

| Stage of disease | Inhibition Absent | Inhibition Present | Total number |
|---|---|---|---|
| Pretreatment | 11 | 8 | 19 |
| Acute phase and relapse | 4 | 16 | 20 |
| Remission | 6* | 1† | 7 |

* On chemotherapy only.

† On immunotherapy.

FIG. 2.—Inhibitory effect of AML serum on PHA response of autologous washed lymphocytes measured by $^{125}$IUdR uptake. NHS = normal human serum.

when AR received immunotherapy with allogeneic blast cells she developed antibodies directed against HL–A 10, 12, 13 and W10 and her serum now showed a much more restricted inhibitory pattern, for example, it completely inhibited the PHA (7·2 μg/ml culture) response of allogeneic lymphocytes with the HL–A phenotype 3, 12, Te58, but the response of autologous lymphocytes and lymphocytes of a donor with HL–A phenotype 2, 9, LND were normal.

The effect of PHA concentration on inhibition was investigated using 5 sera which gave more than 80% inhibition of the normal lymphocyte PHA (7·2 μg/ml culture) response. The values for each serum at 7·2 μg PHA/ml culture were 6%, 2%, 2%, 3% and 15% of the NHS response, whilst at 21·6 μg PHA/ml culture the values were respectively 106%, 110%, 146%, 56% and 107% of the NHS response at 7·2 μg PHA/ml culture. In all cases the inhibitory effect of the leukaemia sera was reduced or abolished at the higher PHA concentration. Inhibition in sera from patient AR due to HL–A antibodies was also abolished at higher PHA concentrations (21·6 μg/ml culture).

Four leukaemia sera (not containing detectable HL–A antibodies) which strongly inhibited the PHA response were also tested in the 2-way mixed lymphocyte culture with a final concentration in the culture medium of 20%. The $^{125}$IUdR uptakes measured in a 3-hour incubation period after 6 days culture were 30%, 41%, 48% and 62% of the uptakes with similar cultures containing 20% NHS.

*The PHA (7·2 μg/ml culture) reactivity of washed lymphocytes from AML patients*

Before treatment most AML patients had circulating blastoid or other primitive cells and therefore lymphocyte cultures of these patients were almost always contaminated with primitive cells, making comparison with normal lymphocyte cultures difficult. However, the circulating blastoid cell population was substantially reduced following the start of induction chemotherapy. The PHA (7·2 μg/ml culture) responses of washed lymphocytes from these patients could then be compared with the responses of normal lymphocytes. Table II shows that there were no significant differences between the responses of normals and leukaemics.

TABLE II.—*$Log_{10}$ PHA (7·2 μg/ml culture) Response in NHS of Lymphocytes from AML Patients Receiving Chemotherapy*

| | No. of patients | No. of tests | $Log_{10}$ PHA response (mean ± 2 SD) |
|---|---|---|---|
| Induction chemotherapy | 18 | 44 | 4·5471 ± 0·2624 |
| Normal | 10 | 19 | 4·4530 ± 0·3501 |

The PHA (7·2 μg/ml culture) responses of 3 patients, measured repeatedly during induction and consolidation stages of chemotherapy, showed a greater proportion of high responses during the consolidation stage (Fig. 3). The differences between the means of the $log_{10}$ induction responses (4·3677) and the $log_{10}$ consolidation responses (4·8214) were significant ($P = 0·05$).

*The effect of the presence of primitive cells on the* in vitro *PHA (7·2 μg/ml culture) response*

With cultures of lymphocytes obtained from AML patients before treatment there was an inverse relationship between the number of primitive cells (usually myeloblasts) present and the PHA response. When the circulating primitive cell concentration was > 8000/mm$^3$ (>approximately 5 × 10$^6$ primitive cells/0·5 × 10$^6$ lymphocytes/culture), the PHA responses were more than 2 standard deviations below the normal mean value and in many cases were not significantly greater than values obtained in control cultures without PHA. However, when circulating primitive cell concentrations were low (< 8000/mm$^3$ and < approxi-

FIG. 3.—PHA responses (measured by $^{125}$IUdR uptake) of 3 AML patients during induction and consolidation chemotherapy. + patient 1, ○ patient 2, ● patient 3.

FIG. 4.—Effect of induction chemotherapy on the PHA response, measured by $^{125}$IUdR uptake, of pretreatment AML patients. ○ patients with no circulating primitive cells, ◑ patients with less than 8000/mm$^3$ circulating primitive cells, ● patients with more than 8000/mm$^3$ circulating primitive cells.

mately $5 \times 10^6$ primitive cells/0·5 $\times 10^6$ lymphocytes/culture) PHA responses did not usually differ significantly from normal (Fig. 4).

In order to investigate the cause of low responses in cultures containing large numbers of primitive cells 3 types of culture were set up: (1) in sealed tubes (standard culture conditions); (2) in loose capped tubes (cotton wool plug); (3) in sealed tubes with a change of medium after 24 and 48 hours of culture (Table III).

TABLE III.—*Effect of Experimental Conditions on PHA* (7·2 *μg/ml Culture*) *response in Cultures Containing a High Proportion of primitive Cells*

| Individual | Cell count/culture | PHA concn (μg/ml culture) | Culture conditions: Sealed tube | Sealed tube medium change at 24 and 48 h* | Loose capped tube cotton-wool plug |
|---|---|---|---|---|---|
| | | | dpm/0·5 × 10⁶ lymphocytes/h | | |
| AML patient No. 1 | Lymphocyte: 0·72 × 10⁶ | 0 | 4155 | 7504 | 15458 |
| | Primitive cell: 13·4 × 10⁶ | 7·2 | 5583 | 18711 | 32569 |
| AML patient No. 2 | Lymphocyte: 0·71 × 10⁶ | 0 | 1866 | 12472 | 7593 |
| | Primitive cell: 5·5 × 10⁶ | 7·2 | 3246 | 21973 | 12115 |
| Normal volunteer No. 1 | Lymphocyte: 0·53 × 10⁶ | 0 | 530 | 532 | 731 |
| | | 7·2 | 21746 | 26373 | 10993 |
| Normal volunteer No. 2 | Lymphocyte: 0·65 × 10⁶ | 0 | 706 | 748 | 650 |
| | | 7·2 | 40938 | 37108 | 3543 |

* 1 ml of supernatant was removed and replaced by 1 ml of TC199 medium/20% serum containing PHA (7·2 μg/ml) or without PHA for control tubes.

In the loose capped and medium change AML cultures the control responses (no PHA added) were greater than those measured in sealed tubes. The pH remained in the range 7·2–7·4 in loose capped cultures and was restored to this pH range in cultures with medium change at 24 and 48 hours, but in contrast the pH consistently fell below 7·1 in sealed tube cultures. Significantly, no response occurred in sealed tube cultures whilst a response to PHA could be measured in both types of culture with modified conditions.

In cultures containing normal lymphocytes the PHA responses in sealed tube and medium change cultures were similar (pH in the culture period in the range 7·1–7·3) whereas in loose capped cultures the PHA response was markedly reduced and the pH increased to 7·9–8·1.

### DISCUSSION

Sera from the majority of patients with AML inhibited the *in vitro* response to PHA of both autologous lymphocytes and allogeneic normal lymphocytes. This occurred only in the appropriate culture conditions and especially during the acute phase or in relapse. Similarly, mixed lymphocyte reactions were inhibited by some of the same AML sera, further suggesting that *in vivo* immune responses could be impaired by factors present in these sera. These observations may explain the reduced delayed hypersensitivity responses in AML which have been reported by some workers (Hersh *et al.*, 1971; Dupuy *et al.*, 1971).

Inhibitors of the lymphocyte response to PHA occur in sera from patients with a wide variety of clinical conditions including uraemia (Silk, 1967*a*), cancer (Silk, 1967*b*), Hodgkin's disease (Trubovitz, Masek and Del Rosario, 1966), idiopathic steatorrhoea (Winter *et al.*, 1967), syphilis (Levene *et al.*, 1969), multiple sclerosis and cirrhosis (Knowles *et al.*, 1968), ataxia telangiectasia (McFarlin and Oppenheim, 1969), advanced alcoholic cirrhosis (Hsu and Leevy, 1971), and also in normal foetal plasma (Ayoub and Kasakura, 1971), normal pregnancy sera (Walker, Freeman and Harris, 1972) and normal sera containing HL–A antibodies (Ceppellini, 1971). Normal serum itself, when present in cultures at high concentrations, is inhibitory (Cooperband *et al.*, 1967). Thus, many different inhibitory factors may be involved—both specific, for example HL–A antibodies, but probably mostly nonspecific. In one example an $\alpha$ globulin fraction separated and concentrated from NHS was found to be immunosuppressive when added to mitogen stimulated and mixed leucocyte cultures (Cooperband *et al.*, 1969).

Even in cases of AML with a high circulating primitive cell count the maintenance of physiological pH in cultures resulted in a significant PHA response by lymphocytes suspended in allogeneic normal serum. Similarly, in most cases where a serum was inhibitory with autologous lymphocytes a normal response could be measured if the PHA concentration was increased. Our evidence thus demonstrates that circulating PHA responsive cells in treated and untreated AML, even in the presence of high leukaemia blast counts and during intensive chemotherapy, can be induced to respond *in vitro*. Indeed, the lymphocytes of 3 patients with AML studied during consolidation therapy with cyclophosphamide and thioguanine had an apparently increased PHA response, an observation which may be explained by the relative susceptibility to cyclophosphamide of " B " lymphocytes and a consequent increase in the relative numbers of " T " cells responsive to PHA.

These studies show that the inhibitory effect of leukaemia serum and the apparent inhibition due to changes in culture conditions when large numbers of blastoid cells are present must be considered when *in vitro* lymphocyte reactivity in AML is measured.

*In vivo* it may be important to distinguish between intrinsic cellular defects and the effect of circulating inhibitory factors. The absence in most cases of inhibitory factors during remission might indicate normal " T " lymphocyte reactivity in these patients, whilst the prompt induction of HL–A alloantibodies in a patient receiving immunotherapy indicates that at least some aspects of " B " cell function were effective in this patient during remission.

Disturbance of the proportions of various lymphocyte classes and the presence of inhibitors, whether specific immunoglobulins or nonspecific and heterogeneous, may be secondary concomitants of leukaemia and chemotherapy which interfere with a specific immunological response. Thus, cellular immunosuppressive activity might prove to be of importance in the maintenance and progress of the disease.

Studies are now in progress which will relate the clinical response of patients with AML to the results of these immunological tests and will be reported elsewhere.

We are deeply indebted to our haematological colleagues for their assistance in obtaining blood samples. Our thanks are due particularly to Mrs S. R. Woods and also to Mr and Mrs J. Wentzel for their technical assistance and to Mrs S. Brooks and Mrs E. Quinn for secretarial assistance. We are grateful to the Leukaemia Research Fund, the Medical Research Council, United Manchester Hospitals' Board of Governor's Research Grants Committee and G. D. Searle & Co. for financial assistance and to the Department of Medical Illustration, Manchester Royal Infirmary, for their help with the illustrations.

### REFERENCES

ASTALDI, G., MASSIMO, L., AIRO, R. & MORI, P. G. (1966) Phytohaemagglutinin and Lymphocytes from Acute Lymphocytic Leukaemia. *Lancet*, i, 1265.

AYOUB, J. & KASAKURA, S. (1971) *In vitro* Response of Foetal Lymphocytes to PHA and a Plasma Factor which Suppresses the PHA Response of Adult Lymphocytes. *Clin. & Exp. Immunol.*, **8**, 427.

BARDARE, M., ACCORSI, A., APOLLONIO, T. & CAREDDU, P. (1969) Blastigenesi Linfocitaria *in vitro* da PHA in Bambini affetti da Leucemia Acuta. *Minerva pediat.*, **21**, 1019.

CEPPELLINI, R. (1971) Old and New Facts and Speculations about Transplantation Antigens of Man. *Prog. Immunol.* In the press.

COOPERBAND, S. R., GREEN, J. A., KENNEDY, M. A. & GRANT, M. M. (1967) Dissociation and Inhibition of the Stimulatory Effect of Phytohaemagglutinin on Protein and DNA Synthesis in Human Lymphocyte Cultures. *Nature, Lond.*, **214**, 1240.

COOPERBAND, S. R., DAVIS, R. C., SCHMID, K. & MANNICK, J. A. (1969) Competitive Blockade of Lymphocyte Stimulation by a Serum Immunoregulatory Alpha Globulin (IRA) *Transplant Proc.*, **1**, 1.

COULSON, A. S. & CHALMERS, D. G. (1967) Response of Human Blood Lymphocytes to Tuberculin PPD in Tissue Culture. *Immunology*, **12**, 417.

CRAIG, A. W., GARRETT, J. V. & JACKSON, S. M. (1969) Quantitation of Lymphocyte Transformation using Radioactive Iododeoxyuridine. *J. clin. Path.*, **22**, 558.

DOENHOFF, M. J., DAVIES, A. J. S., LEUCHARS, E. & WALLIS, V. (1971) The Thymus and Circulating Lymphocytes of Mice. *Proc. R. Soc.* Series B. *Biological Sciences*, **176**, 69.

DUPUY, J. M., KOURILSKY, F. M., FRADELIZZI, D., FEINGOLD, N., JACQILLAT, C., BERNARD, J. & DAUSSET, J. (1971) Depression of Immunologic Reactivity of Patients with Acute Leukaemia. *Cancer, N.Y.*, **27**, 323.

FITZGERALD, M. G. (1971) The Establishment of a Normal Human Population Dose–Response Curve for Lymphocytes Cultured with PHA (Phytohaemagglutinin). *Clin. & Exp. Immunol.*, **8**, 421.

FREEMAN, C. B., WALKER, J. S., COCKING, H. & HARRIS, R. (1973) Serum Inhibitors in Acute Leukaemia. *Proc.* 1972 *Cancer Week* (Int. Coll. CNRS). In the press.

FRIDMAN, W. H. & KOURILSKY, F. M. (1969) Stimulation of Lymphocytes by Autologous Leukaemic Cells in Acute Leukaemia. *Nature, Lond.*, **224**, 277.

HARRIS, R. (1973) Leukaemia Antigens and Immunity in Man. *Nature, Lond.*, **241**, 95.

HARRIS, R., WENTZEL, J., COCKING, H., DODSWORTH, H. & UKAEJIOFO, E. O. (1970) Errors in Allograft Donor Typing: A Modified Microcytotoxic Test. *Histocompatibility Testing.* Copenhagen: Munksgaard.

HERSH, E. M., WHITECAR, J. P., MCCREDIE, K. B., BODEY, G. P. & FREIREICH, E. J. (1971) Chemotherapy, Immunocompetence, Immunosuppression and Prognosis in Acute Leukaemia. *New Engl. J. Med.*, **285**, 1211.

HSU, C. S. C. & LEEVY, C. M. (1971) Inhibition of PHA-stimulated Lymphocyte Transformation by Plasma from Patients with Advanced Alcoholic Cirrhosis. *Clin. & Exp. Immunol.*, **8**, 749.

JONES, L. H., HARDISTY, R. M., WALLS, D. G. & KAY, H. E. M. (1971) Lymphocyte Transformation in Patients with Acute Lymphoblastic Leukaemia. *Br. med. J.*, iv, 329.

KNOWLES, M., HUGHES, D., CASPARY, E. A. & FIELD, E. J. (1968) Lymphocyte Transformation in Multiple Sclerosis: Inhibition of Unstimulated

Thymidine Uptake by a Serum Factor. *Lancet*, ii, 1207.

LEVENE, G. M., TURK, J. L., WRIGHT, D. J. M. & GRIMBLE, A. G. S. (1969) Reduced Lymphocyte Transformation due to a Plasma Factor in Patients with Active Syphilis. *Lancet*, ii, 246.

MCFARLIN, D. E. & OPPENHEIM, J. J. (1969) Impaired Lymphocyte Transformation in Ataxia Telangiectasia in Part Due to a Plasma Inhibitory Factor. *J. Immun.*, **103**, 1212.

SILK, M. R. (1967*a*) The Effect of Uremic Plasma on Lymphocyte Transformation. *Invest. Urol.*, **5**, 195.

SILK, M. R. (1967*b*) Effect of Plasma from Patients with Carcinoma on *in vitro* Lymphocyte Transformation. *Cancer, N.Y.*, **20**, 2088.

TRUBOWITZ, S., MASEK, B. & DEL ROSARIO, A. (1966) Lymphocyte Response to Phytohaemagglutinin in Hodgkin's Disease, Lymphocytic Leukaemia and Lymphosarcoma. *Cancer, N.Y.*, **19**, 2019.

VIZA, D., BERNARD-DEGANI, O., BERNARD, CL. & HARRIS, R. (1969) Leukaemia Antigens. *Lancet*, ii, 493.

WALKER, J. S., FREEMAN, C. B. & HARRIS, R. (1972) Lymphocyte Reactivity in Pregnancy. *Br. med. J.*, iii, 469.

WINTER, G. C. B., MCCARTHY, C. F., READ, A. E. & YOFFEY, J. M. (1967) Development of Macrophages in PHA Cultures of Blood from Patients with Idiopathic Steatorrhoea and Cirrhosis. *Br. J. exp. Path.*, **48**, 66.